# Haplotypes of the *HLA-G* 3’ Untranslated Region Respond to Endogenous Factors of HLA-G+ and HLA-G- Cell Lines Differentially

**DOI:** 10.1371/journal.pone.0169032

**Published:** 2017-01-03

**Authors:** Isabelle Poras, Layale Yaghi, Gustavo Martelli-Palomino, Celso T. Mendes-Junior, Yara Costa Netto Muniz, Natalia F. Cagnin, Bibiana Sgorla de Almeida, Erick C. Castelli, Edgardo D. Carosella, Eduardo A. Donadi, Philippe Moreau

**Affiliations:** 1 Commissariat à l’Energie Atomique et aux Energies Alternatives, Institut des Maladies Emergentes et des Thérapies Innovantes, Service de Recherches en Hémato-Immunologie, Hôpital Saint-Louis, Paris, France; 2 Université Paris-Diderot, Sorbonne Paris-Cité, UMR_E5, Institut Universitaire d’Hématologie, Hôpital Saint-Louis, Paris, France; 3 Lebanese University, School of Medicine, Hadath, Lebanon; 4 Programa de Pós-graduação em Ciências da Saúde, Centro de Ciências da Saúde. Universidade Federal do Rio Grande do Norte, Natal, Rio Grande do Norte, Brasil; 5 Departamento de Química, Faculdade de Filosofia, Ciências e Letras de Ribeirão Preto, Universidade de São Paulo, Ribeirão Preto, São Paulo, Brazil; 6 Departamento de Biologia Celular, Embriologia e Genética, Centro de Ciências Biológicas, Universidade Federal de Santa Catarina, Florianopolis, Santa Catarina, Brasil; 7 Department of Genetics, Ribeirão Preto Medical School, University of São Paulo, Ribeirão Preto, São Paulo, Brazil; 8 Divisão de Imunologia Clínica, Departamento de Clínica Médica, Faculdade de Medicina de Ribeirão Preto, Universidade de São Paulo, Ribeirão Preto, São Paulo, Brasil; 9 Department of Pathology, School of Medicine, Universidade Estadual Paulista, Unesp, Botucatu, São Paulo, Brazil; National Center for Toxicological Research, UNITED STATES

## Abstract

The immune checkpoint HLA-G prevents maternal rejection of the fetus and contributes in cancer invasion and acceptance of allografts. The 5’ and 3’ regulatory regions of the *HLA-G* gene are polymorphic and balancing selection probably maintains this variability. It is proposed that nucleotide variations may affect the level of HLA-G expression. To investigate this issue we aimed to analyze how haplotypes of the 3’ untranslated region (3’UTR) with highest worldwide frequencies, namely UTR-1, UTR-2, UTR-3, UTR-4, UTR-5, UTR-18 and UTR-7, impact the expression of a luciferase reporter gene *in vitro*. Experiments performed with the HLA-G positive cell lines JEG-3 (choricarcinoma) and FON (melanoma), and with the HLA-G negative cell lines M8 (melanoma) and U251MG (glioblastoma) showed that the *HLA-G* 3’UTR polymorphism influences the response to endogenous cellular factors and may vary according to the cell type. UTR-5 and UTR-7 impact the activity of luciferase the most whereas UTR-2, UTR-3, UTR-4, and UTR-18 have intermediate impact, and UTR-1 has the lowest impact. These results corroborate the previous associations between amounts of plasma sHLA-G levels and 3’UTR haplotypes in healthy individuals and reinforce that 3’UTR typing may be a predictor of the genetic predisposition of an individual to express different levels of HLA-G.

## Introduction

HLA-G is a major immune checkpoint molecule. The earliest studies on HLA-G demonstrated that this non-classical HLA is expressed on the cell surface of cytotrophoblasts, where it plays a key function in maternal-fetal tolerance [1]. HLA-G exerts its inhibitory actions on immunocompetent cells by primarily interacting with the ILT-2 / LILRB1 [2] and ILT-4 / LILRB2 [3] receptors. In contrast to normal conditions in which the expression of HLA-G is very restricted to few tissues, ectopic HLA-G expression is common in pathological situations such as cancer, and favors the tumor escape from the patient’s immune surveillance. In the case of transplantation, HLA-G favors the acceptance of allografts [4]. It is noteworthy that the levels of HLA-G expression vary across individuals [5], suggesting that the 53 identified *HLA-G* alleles (IPD-IMGT/HLA database, version 3.24.0.1), and their associated regulatory regions might respond to a specific microenvironment differentially. Nevertheless, the mechanisms underlying the normal and ectopic HLA-G expression are partially elucidated. Regulation of *HLA-G* expression differs from regulation of classical *HLA-class I* genes [6]. HLA-G expression is regulated both at the transcriptional and post-transcriptional levels [7] and involves at least four alternative transcripts for membrane-bound HLA-G forms (HLA-G1 to HLA-G4) [8, 9] and three alternative transcripts for soluble forms (HLA-G5 to HLA-G7) [10–12].

A 5’ regulatory region extending at least 1.4 kb from the ATG translation initiation site controls *HLA-G* gene transcription. This region contains a Locus Control Region [13] and functional sites to which several transcription factors bind, including CREB-1, HSF-1, IRF-1, PR [6] and the repressors, RREB-1 [14] and GLI-3 [15]. The DNA methylation status of the *HLA-G* locus is also important to control the *HLA-G* transcriptional activity [16]. To date at least 35 single nucleotide variations (SNV) have been listed in the worldwide population, which define a total of 68 haplotypes of the 1.4 kb regulatory region [17–21]. Variable sites might influence the expression of HLA-G by affecting the differential binding of some transcription factors and might influence the methylation of DNA [7].

**Fig 1:**
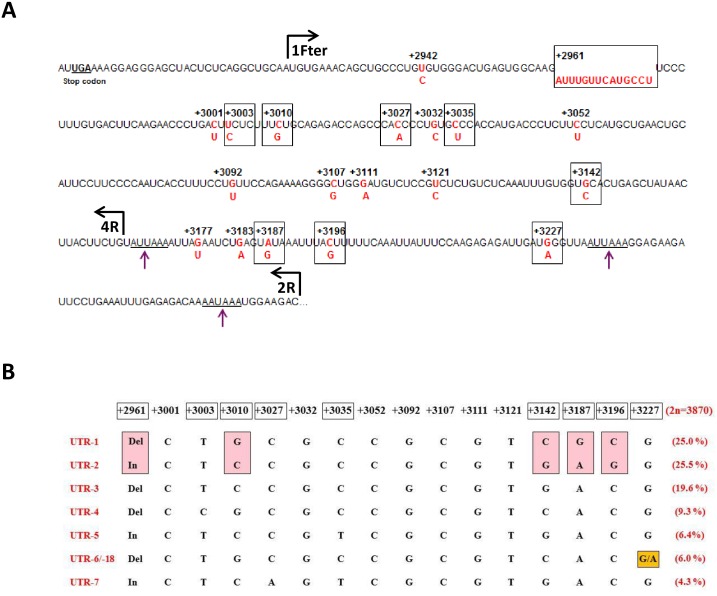
*HLA-G* 3’UTR polymorphisms. (A) Variations in *HLA-G* mRNA (red) along the 3’UTR. The less frequent variants are positioned below the most frequent one in the nucleotide sequence. Polymorphic sites are framed in bold when the frequency of the minor allele worldwide is higher than 1%. Purple single arrows point to the three predicted polyadenylation signals. Vertical lines with horizontal arrows indicate the 5’ end of the *HLA-G* nucleotide sequences for 1Fter, 2R and 4R primers used in pMIR-REPORT^™^ constructions. (B) Sequence comparison of the most frequent 3’UTR haplotypes (21 worldwide populations; 2n = 3870) [25] analyzed in the present study. Frequencies are indicated on the right part. UTR-1 and UTR-2 differ at 5 positions (pink rectangles). UTR-6 and UTR-18 (analyzed in the present work) only differ at one position and depending on the UTR typing strategy that was used (i.e., targeting or not +3227 SNP), UTR-18 could be confused with UTR-6 in several studies.

On the other hand, published data has pointed out that the 3’ untranslated region (UTR) shared by the HLA-G1 to HLA-G6 alternative transcripts (~ 370 bases, Fig 1A) has a crucial role in modulating the expression of HLA-G. A first important finding is that this segment is targeted by six already identified microRNAs, namely miR-148a, miR-148b, miR-152 [22], miR-133a [23], miR-628-5p and miR-548q [24] which can downregulate HLA-G expression. Another finding is that the 3’UTR is polymorphic, with a genetic diversity likely influenced by balancing selection as observed in the case of the *HLA-G* promoter region. Worldwide analyses have recorded 18 single nucleotide polymorphisms (SNP) and a 14 bp insertion/deletion (InDel; rs3711944629). A total of 44 haplotypes have been identified, but only nine variable sites exhibit frequency of the minor allele higher than 1% and could therefore be considered as true polymorphisms [18, 25]. UTR-1, UTR-2, UTR-3, UTR-4, UTR-5, UTR-6/-18, and UTR-7 are the most frequent 3’UTR haplotypes at the global level and account for more than 96% of all the reported haplotypes (Fig 1B) [25]. UTR-1 (~25%) and UTR-2 (~25%), which differ at five positions, are the predominant haplotypes, followed by UTR-3. Some 3’UTR polymorphisms have been associated with the magnitude of HLA-G expression. The presence of the 14-nucleotide sequence (14 bp In) has been associated with a unique alternative splicing pattern where 92 bases are removed from the 3’UTR of the mature mRNAs in a subpopulation of transcripts [26]. The smaller transcripts are more stable than the transcripts that retain the 92-base segment [27]. Moreover, except for a study that showed higher expression of HLA-G1 on the cell surface of HLA-G-transfected cells [28], the 14 bp In allele has been associated with lower *HLA-G* mRNA and lower HLA-G cell surface expression of first trimester trophoblasts [29], as well as with lower levels of soluble HLA-G (sHLA-G) in blood plasma, serum and seminal plasma [30–32]. Otherwise, a polymorphic site at position +3142 (rs1063320) [22] consists of either in a Guanine (G) with a world frequency of 58.8% or in a Cytosine (C) [25]. Interestingly, the seed region of the microRNAs miR-148a,b and miR-152 targets the G-C SNV in the *HLA-G* mRNA. Calculation of the minimum free energy (MFE), has predicted that binding of each microRNA to the Guanine allele is more stable than binding of each microRNA to the Cytosine allele [22]. However, *in vitro* experiments to demonstrate it have afforded controversial results [33]. Finally, it was demonstrated *in vitro* that the presence of an Adenine (A) instead of a Guanine (G) at +3187 location (rs9380142) affects mRNA stability, leading to a shorter *HLA-G* mRNA half-life [34]. The proximity of the polymorphic site to an AU-rich motif might decrease mRNA stability in the presence of an increased number of Adenines in this region. Therefore, given the 14 bp InDel, the +3142 G-C and the +3187 A-G polymorphic sites, it was hypothesized that 3’UTR haplotypes containing the 14 bp In, +3142 G and +3187 A variants might be associated with low production of HLA-G, whereas 3’UTR haplotypes carrying the 14 bp Del, +3142 C and +3187 G variants might be associated with high expression of HLA-G [35].

To investigate how polymorphic sites along the 3’UTR impact on the level of sHLA-G expression, we have recently genotyped healthy individuals from Brazil and France and measured the amounts of plasma sHLA-G (n = 259) [5]. We were able to distinguish three groups of diplotypes associated with higher, lower and medium levels of sHLA-G. The group that produced higher levels of sHLA-G consisted mainly of diplotypes containing UTR-1, which exhibits all variants previously associated with high HLA-G expression (14 bp Del, +3142 C and +3187 G). The group that produced lower levels of sHLA-G consisted mainly of diplotypes containing UTR-5 and UTR-7 which exhibit all variants previously associated with low HLA-G expression and differ by a single position (+3027 C and A, respectively; rs17179101). Interestingly, UTR-2 was classified as a medium producer although it bears the trio of variants found in UTR-5 and UTR-7, and differs only at positions +3027 (compared with UTR-7), +3035 (rs17179108) and +3196 (rs1610696). To understand these previous results further, the present study aims to investigate *in vitro*, using luciferase activity assays, whether *HLA-G* 3’UTR polymorphism influences endogenous factors that could affect mRNA stabilization or degradation (including RNA-binding proteins and microRNAs). We used four different cell lines that express HLA-G (choriocarcinoma JEG-3 and melanoma FON) or not (Melanoma M8 and glioma U251MG) to focus on *HLA-G* 3’UTR haplotypes that are frequently found worldwide, namely UTR-1, UTR-2, UTR-3, UTR-4, UTR-5, UTR- 18 and UTR-7 (Fig 1B).

## Materials and Methods

### Human cell line cultures

Cell lines expressing HLA-G (JEG-3, FON) or not HLA-G (M8, U251MG) at the cell surface [36, 37] were cultured at 37°C under 5% CO_2_. The JEG-3 choriocarcinoma cell line (ATCC, Manassas, VA, USA) and the U251MG glioma cell line (provided by Dr. Heinz Wiendl, University of Münster) were cultivated in DMEM with Glutamax-I (Invitrogen, Cergy Pontoise, France). The M8 and FON melanoma cell lines [36] were maintained in RPMI-1640 medium (Sigma-Aldrich, St. Quentin Fallavier, France) with 2 mM L-glutamin (Life Technologies, ThermoFisher Scientific, Villebon Sur Yvette, France). All the cells were cultured in medium supplemented with 10% heat-activated fetal calf serum (Sigma-Aldrich), 250 μg/L fungizone (Invitrogen, ThermoFisher Scientific) and 10 mg/L gentamicin (Invitrogen, ThermoFisher Scientific).

### *HLA-G* transcriptional activity and *HLA-G* 3’UTR typing of JEG-3, FON, M8 and U251MG cell lines

RNAs were extracted with TRIzol^®^ reagent (Invitrogen) according to the manufacturer’s protocol, reverse-transcribed with Moloney murine leukemia virus reverse transcriptase (Invitrogen) and amplified in triplicate by duplex Real-Time PCR targeting *HLA-G* exon 5 and GAPDH as an endogenous control, as previously described [16]. Quantification was performed relative to amounts of HLA-G transcripts in HLA-G-positive JEG-3 cells by using the comparative C_T_ method [16]. S1 Fig illustrates representative HLA-G transcriptional activities of JEG-3, FON, M8, and U251MG cells.

Genomic DNAs were extracted and purified using the standard phenol-chloroform procedure. The *HLA-G* 3'UTRs were isolated by PCR using the HLAG8R (5’-GTCTTCCATTTATTTTGTCTCT-3’) and HLAG8F (5’-TGTGAAACAGCTGCCCTGTGT-3’) primers [5]. Each amplification product was directly sequenced with the HLAG8R primer in an automatic sequencer ABI PRISM 310 Genetic Analyzer (Applied Biosystems, Foster City, CA), with the BigDye terminator v3.1 cycle sequencing kit (Applied Biosystems). The haplotypes for each sample were determined by two independent computational methods: the expectation-maximization (EM) algorithm implemented by the PL-EM software [38] and a coalescence-based method implemented by the PHASE v2 software [39]. We identified UTR-2/UTR-7 haplotypes in JEG-3 cells, UTR-2/UTR-2 haplotypes in FON cells, UTR-1/UTR-2 haplotypes in M8 cells and UTR-1/UTR-4 haplotypes in U251MG cells.

### Reporter constructs

For each 3’UTR haplotype (UTR-1, UTR-2, UTR-3, UTR-4, UTR-5, UTR-18, and UTR-7) two constructions were performed with the pMIR-REPORT^™^ vector (Ambion^®^, ThermoFisher Scientific): the putative *HLA-G* polyadenylation signals were kept (1Fter-2R primer set used) or not (1Fter-4R primer set used), thereby maintaining or deleting the polymorphic sites at positions +3187, +3196, and +3227 (Fig 1A). Briefly, plasmids were digested by Hind III and Spe I (Fermentas, ThermoFisher Scientific), within the multiple cloning site located at position 3’ relative to the luciferase reporter gene and then ligated (T4 DNA ligase; New England Biolabs, Evry, France) with a Hind III and Spe I-digested PCR product encompassing the 3’UTR fragments. Amplifications were carried out on human genomic DNAs that had been genotyped in a previous study [5], by using the forward primer 1Fter (5’-TTA**ACTAGT**AGTGTGAAACAGCTGCCCT) with Spe I site (highlighted in boldface) and the reverse primers 2R (5’-TTA**AAGCTT**GTCTTCCATTTATTTTGTCTCTCAA) or 4R (5’-TTA**AAGCTT**CAGAAGTAAGTTATAGCTCAGTG) with Hind III site (highlighted in boldface). Site-directed mutagenesis targeting +3035 SNP were carried out by Proteogenix (Schiltidheim, France). The constructions were validated by means of nucleotide sequencing (GATC Biotech SARL, Mulhouse, France).

### Transient transfection and luciferase activity assays

Cells with 70% confluence were co-transfected with 2.5 μg of each pMIR-REPORT^™^ construction and 0.05 μg of the pRL-TK Renilla Luciferase reporter vector (Promega, Charbonnières-les-Bains, France) by using the Lipofectamin 2000 reagent (Invitrogen, ThermoFisher Scientific). After 48 h of transfection, the reporter assays were carried out with the Dual Luciferase Reporter Assay System (Promega) according to the manufacturer’s instructions. Aliquots of cell lysates were transferred to a 96-well microplate and successively incubated with the Firefly Luciferase and Renilla Luciferase substrates. The luminescence signal was recorded with the FluoStar Optima Plate reader (BMG Labtech). Firefly luciferase values were first normalized to those of Renilla luciferase values, and then to luciferase expression of the empty pMIR-REPORT^™^ (assigned a value of 1) for each of the 3’UTR constructions (S1 Table). Assays were run in duplicates from at least three independent experiments.

### Statistical analysis

The Kolmogorov-Smirnov normality test was used to decide whether the analysis of the modulation of HLA-G expression levels, and their respective associations with the 3’UTR haplotypes, should be conducted with parametric or non-parametric tools. Because the data did not meet the assumptions of parametric tests, non-parametric analyses were accomplished. The two-tailed Mann-Whitney test was used to compare data between two sample groups, and the Kruskal-Wallis test followed by the Dunn’s multiple comparison post-test were used to compare data among three or more sample groups. These analyses were performed with the programs PASW Statistics (17.0.2) (SPSS Inc, Chicago, IL, USA) and GraphPad InStat 3.06 (GraphPad Software Inc, San Diego, CA, USA). For all instances, a 5% significance level was taken into account. All the graphs were constructed with the Minitab 17 Statistical Software (Minitab Inc, State College, PA, USA).

## Results

### The 3’UTR fragment upstream +3165 primarily influences *HLA-G* post-transcriptional regulation

**Fig 2:**
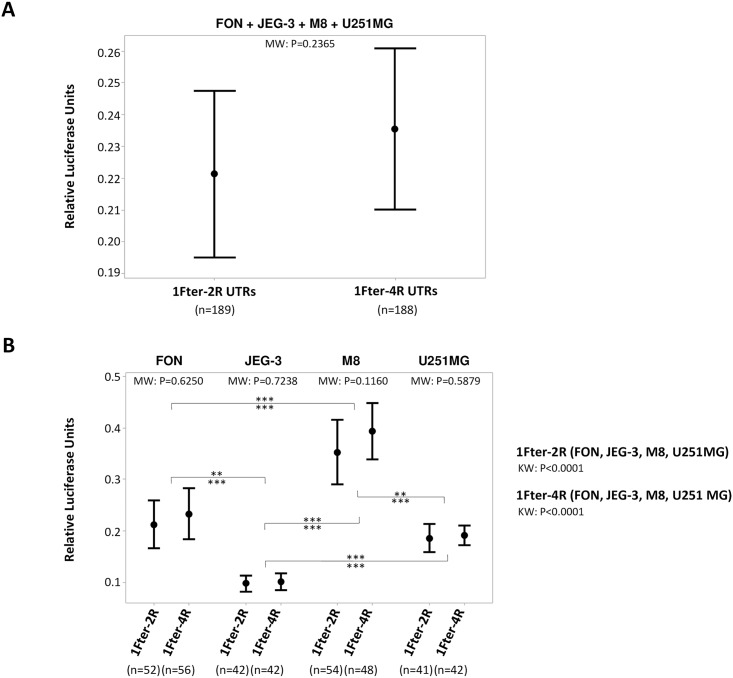
Modulation of luciferase reporter gene expression in FON+, JEG-3, M8 and U251MG cells considering the constructions 1Fter-2R *vs* 1Fter-4R, is not statistically different, regardless of the haplotypes. Points indicate the means and vertical bars indicate the 95% confidence interval. The number of experiments considering each 1Fter construction is indicated between parentheses. MW: Mann-whitney test; KW: Kruskal-Wallis test. (A) Modulation of luciferase gene expression in the four cell lines combined. (B) Modulation of luciferase gene expression in each cell line independently. P values for Dunn’s multiple comparison test are indicated above and under the horizontal bar for the 1Fter-2R and 1Fter-4R constructions respectively (** p value < 0.01; *** p value < 0.001).

Considering the UTR-1, UTR-2, UTR-3, UTR-4, UTR-5, UTR-18 and UTR-7 haplotypes of *HLA-G* 3’UTR segment, we first investigated the influence of the absence or the presence of the three putative polyadenylation (poly-A) signals identified in this region, on the expression of the luciferase reporter gene. We measured it with constructions 1Fter-4R (absence of the fragment containing the *HLA-G* poly-A signals) and 1Fter-2R (presence of the *HLA-G* poly-A signals) (Fig 1A) transfected in HLA-G+ cells (JEG-3, FON) and HLA-G- cells (M8, U251MG). The global expression of luciferase was downregulated as compared with the empty vector and there was no statistically significant difference between the two types of constructions if the four cell lines were considered together (*P* = 0.2365) or each cell line was considered separately (*P* = 0.6250 for FON+, *P* = 0.7238 for JEG3, *P* = 0.1160 for M8, and *P* = 0.5879 for U251MG) (Fig 2A and 2B).

These results suggest that the segment downstream position +3165 up to position +3280 may not influence the action of endogenous factors of each cell line and that the location of polyadenylation signals presumably do not affect this action.

### *HLA-G* 3’UTR polymorphism influences the response to cellular endogenous factors

To analyze the impact of 3’UTR polymorphism *in vitro* we first considered the 14 bp InDel in both construction types (1Fter-4R and 1Fter-2R) in the four cell lines in combination or in each cell line separately. We found that luciferase gene expression was more intensely downregulated by UTRs with the 14 bp insertion (UTR-2, UTR-5, and UTR-7) and less intensely downregulated by UTRs with the 14 bp deletion (UTR-1, UTR-3, UTR-4, and UTR-18) (Table 1). The exception was that the activity of luciferase decreased in M8 (HLA-G-) cells regardless of the 14 bp insertion or deletion (Table 1).

**Table 1:** Impact of the 14 bp InDel on the luciferase reporter gene expression when both construction types (1Fter-4R and 1Fter-2R primers) were considered in each cell line independently or in the four cell lines in combination.

	FON	JEG-3	M8	U251MG	F+J+M+U
**Median 14 bp In**	0.0832 (46)	0.0655 (36)	0.2957 (46)	0.1324 (36)	0.1246 (164)
**Median 14 bp Del**	0.2905 (62)	0.1213 (48)	0.3284 (56)	0.2045 (47)	0.2087 (213)
**MW**	*P* < 0.0001	*P* < 0.0001	*P* = 0.1149	*P* < 0.0001	*P* < 0.0001

MW: The two-tailed Mann-Whitney test was used to compare the haplotype group exhibiting the 14 bp insertion (UTR-2, UTR-5, and UTR-7) and the haplotype group having the 14 bp deletion (UTR-1, UTR-3, UTR-4, and UTR-18). Median Luciferase expression (RLU) is downregulated more intensively by UTRs with 14 bp insertion. (): number of experiments.

**Fig 3:**
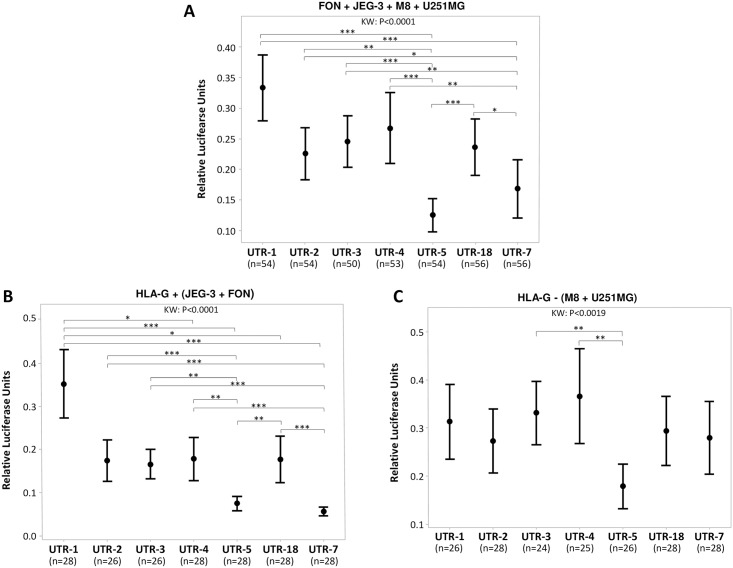
Significant differences in the modulation of the expression of the luciferase reporter gene in FON+, JEG-3, M8 and U251MG cells considering each 3’UTR haplotype independently, with the 1Fter-2R and 1Fter-4R constructions combined. Points indicate the means and vertical bars indicate the 95% confidence interval. The number of experiments considering each 3’UTR haplotype is indicated between parentheses. KW: Kruskal-Wallis test; * p value <0.05; ** p value < 0.01; *** p value < 0.001. (A) Modulation of luciferase expression in the four cell lines combined. (B) Modulation of luciferase expression in the HLA-G+ (FON + JEG-3) cell lines combined. (C) Modulation of luciferase expression in the HLA-G- (M8 + U251MG) cell lines combined. In (A) and (B) luciferase expression is significantly more intensely downregulated by UTR-5 and UTR-7 than by other haplotypes.

Regarding each haplotype with both constructions in the four cell lines, we observed statistically significant differences in the modulation of luciferase expression (*P*<0.0001) which was downregulated more intensely by UTR5 and UTR7 and less intensely by UTR-1, and also by UTR-2, UTR-3, UTR-4 and UTR-18 (Fig 3A). In agreement with these findings, simultaneous comparison of the seven 3’UTR haplotypes revealed significant differences in the modulation of luciferase expression when both constructions were considered in either HLA-G+ cells (JEG-3 and FON, *P*<0.0001) or HLA-G- cells (M8 and U251MG, *P* = 0.0019) (Fig 3B and 3C).

In addition, pairwise analysis considering each haplotype with both constructions in HLA-G+ cells revealed significant differences in all the pairs involving UTR-5 or UTR-7 *versus* UTR-1, UTR-2, UTR-3, UTR-4 or UTR-18 whereas significant differences emerged solely in pairs involving UTR-5 *versus* UTR-3 and UTR-5 *versus* UTR-4 when HLA-G- cells are considered (Table 2). Finally, given each cellular background, pairwise analysis showed significant differences (*P*<0.05) in 20 pairs. Eighteen of the pairs involved UTR-5 or UTR-7, which again indicated that the luciferase expression was more intensely downregulated by UTR-5 and UTR-7 than by other UTRs (Table 2).

**Table 2:** Modulation of the expression of the reporter gene luciferase by 3’UTR haplotypes in four cell lines expressing HLA-G or not.

	HLA-G+					HLA-G-					
Cell Lines	JEG-3		FON		Both	M8		U251MG		Both	All
Constructions	1Fter2R	1Fter4R	1Fter2R	1Fter4R	Both	1Fter2R	1Fter4R	1Fter2R	1Fter4R	Both	Both
**Kruskal-Wallis**	<0.0001	0.0083	<0.0001	<0.0001	<0.0001	0.0292	0.6086	<0.0001	0.0057	0.0019	<0.0001
**UTR-1 vs. UTR-2**	-	-	-	-	-	-	-	-	-	-	-
**UTR-1 vs. UTR-3**	-	-	-	<0.05	-	-	-	-	-	-	-
**UTR-1 vs. UTR-4**	-	-	<0.01	-	<0.05	-	-	-	-	-	-
**UTR-1 vs. UTR-5**	<0.001	-	<0.001	<0.001	<0.001	-	-	-	-	-	<0.001
**UTR-1 vs. UTR-18**	-	-	-	-	<0.05	-	-	-	-	-	-
**UTR-1 vs. UTR-7**	<0.001	<0.05	<0.001	<0.001	<0.001	-	-	-	-	-	<0.001
**UTR-2 vs. UTR-3**	-	-	-	-	-	-	-	-	-	-	-
**UTR-2 vs. UTR-4**	-	-	-	-	-	-	-	-	-	-	-
**UTR-2 vs. UTR-5**	-	-	<0.05	-	<0.01	-	-	-	-	-	<0.01
**UTR-2 vs. UTR-18**	-	-	-	-	-	-	-	-	-	-	-
**UTR-2 vs. UTR-7**	-	-	<0.01	-	<0.001	-	-	-	-	-	<0.05
**UTR-3 vs. UTR-4**	-	-	-	-	-	-	-	-	-	-	-
**UTR-3 vs. UTR-5**	<0.01	-	-	-	<0.01	-	-	<0.01	-	<0.01	<0.001
**UTR-3 vs. UTR-18**	-	-	-	-	-	-	-	-	-	-	-
**UTR-3 vs. UTR-7**	<0.01	-	-	-	<0.001	-	-	-	<0.05	-	<0.01
**UTR-4 vs. UTR-5**	-	-	-	<0.01	<0.01	-	-	-	-	<0.01	<0.001
**UTR-4 vs. UTR-18**	-	-	-	-	-	-	-	-	-	-	-
**UTR-4 vs. UTR-7**	-	-	-	<0.001	<0.001	-	-	-	-	-	<0.01
**UTR-5 vs. UTR-18**	-	-	-	-	<0.01	-	-	<0.001	-	-	<0.001
**UTR-5 vs. UTR-7**	-		-		-	-		-		-	-
**UTR-18 vs. UTR-7**	-	-	<0.01	-	<0.001	-	-	<0.01	-	-	<0.05

Non-differentiation *P*-values were assessed by the non-parametric Kruskal-Wallis test (followed by the Dunn’s multiple comparison post-test), used to compare data among seven 3’UTR groups in a given cell line.

### The +3035 C-T variable site does not influence the response of *HLA-G* 3’UTR to cellular endogenous factors

**Fig 4:**
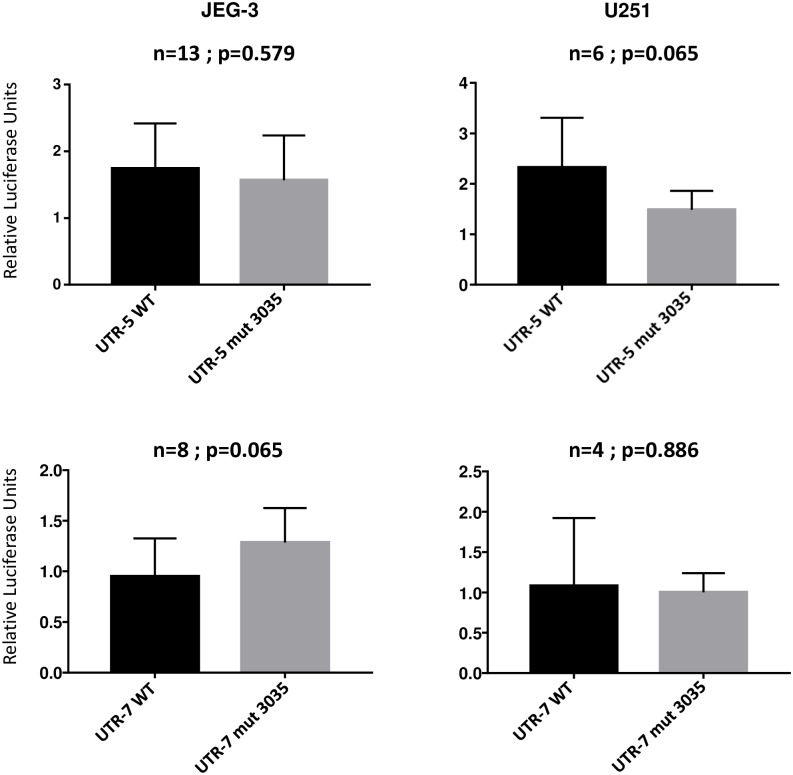
Site-directed mutagenesis replacing a T with a C at position +3035 in UTR-5 and UTR-7 (1Fter-2R constructions) does not significantly influence the levels of luciferase responses. The two-tailed Mann-Whitney test was used to compare the impact of wild type UTR-5 and UTR-7 to the impact of mutated UTR-5 and UTR-7 respectively in JEG-3 and U251MG cells. Data is presented as mean **+/-** SEM.

The response of UTR-2 *in vitro* (intermediate downregulation) differed from the responses of UTR-5 and UTR-7 (strong downregulation) although UTR-2, UTR-5 and UTR7 do share all the variants previously associated with low HLA-G expression. Interestingly both UTR-5 and UTR-7 present a Thymine (T) at position +3035, whereas a Cytosine (C) is present at this same position in UTR-2 and other haplotypes, suggesting that the +3035 SNP should also influence the expression of HLA-G. Therefore, to evaluate whether this variable site directly affects the activity of the luciferase reporter gene in U251MG and JEG-3 cells, we used site-directed mutagenesis to replace a T with a C in UTR-5 and UTR-7 (1Fter-2R constructions). Regardless of the 3’UTR haplotypes, there were no significant differences in luciferase activities when we compared wild and mutated forms (Fig 4). These findings suggest that +3035 SNP alone could not account for the specific responses of UTR-5 and UTR-7.

### The influence of endogenous factors on *HLA-G* 3’UTR varies according to the cell type

**Fig 5:**
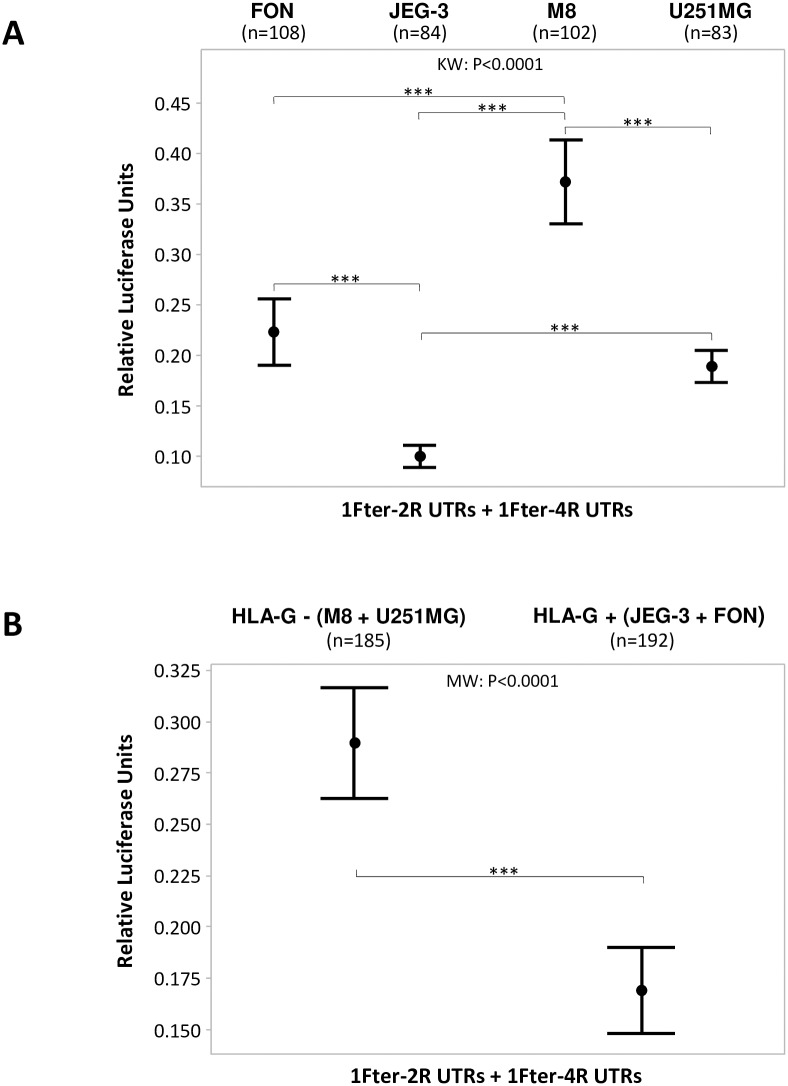
Significant differences in the modulation of the expression of the luciferase reporter gene considering either the cell type or the status of HLA-G expression, with 1Fter-2R and 1Fter-4R constructions and haplotypes combined. Points indicate the mean and vertical bars indicate the 95% confidence interval. The number of experiments considering each cell line or the status of HLA-G expression is indicated between parentheses. MW: Mann-whitney test; KW: Kruskal-Wallis test. (A) Modulation of luciferase gene expression in each cell line separately. The decrease in the luciferase gene activity in U251MG (HLA-G-) and FON cells (HLA-G+) was not statistically different. (B) Modulation of luciferase gene expression in the HLA-G- (M8 + U251MG) cells *vs* HLA-G + (FON + JEG-3) cells combined.

To investigate the association between the status of the HLA-G expression by the cell type and the level of the 3’UTR response to endogenous factors, we considered both constructions 1Fter-4R and 1Fter-2R, irrespective of the haplotype, either in each cell line independently or in HLA-G+ *versus* HLA-G- cells. In Fig 5A, simultaneous comparison of the different cell lines revealed statistically significant differences among them (*P*<0.0001). The exception was the decrease in luciferase activity in U251MG (HLA-G-) and FON cells (HLA-G+) which was statistically identical. Notably, the most and the less intense downregulation of luciferase activity occurred with the JEG-3 cells (HLA-G+) and the M8 cells (HLA-G-), respectively. Otherwise, it is noteworthy that the luciferase activity was more intensely downregulated in HLA-G+ than in HLA-G- cells (*P*<0.0001) (Fig 5B).

## Discussion

The present *in vitro* study provides new insights into how *HLA-G* 3’UTR and its polymorphism affect the level of HLA-G expression. The UTRs of genes have been shown as important regulatory elements of the post-transcriptional regulation of gene expression and they most probably play a pivotal role in the individual’s susceptibility to disease [40]. In particular, genetic variations in these regions can modify interactions with cellular endogenous factors such as RNA-binding proteins and microRNAs [40]. It is noteworthy that in pathological situations where HLA-G is critical to control the patient’s immune system, the magnitude of HLA-G expression may vary depending on the individual [4] [41]. Although there is evidence that *HLA-G* 5’URR polymorphisms may influence the levels of promoter activity [42], investigations into the global impact of 3’UTR polymorphisms are lacking. In the present study we have performed reporter assays of *HLA-G* 3’UTR haplotypes to analyze the differences between alleles in terms of the regulation of mRNA stability by 3’UTR following exposure to endogenous factors of four cell lines expressing HLA-G or not. With regard to the different sites that polymorphisms may affect in *HLA-G* 3’UTR mRNA, we have hypothesized that the modulations in the level of reporter gene expression depends on the 3’UTR haplotype controlling it and the status of HLA-G expression in the cells.

Firstly, our results suggest that the 3’UTR fragment located downstream of the position +3165 up to the position +3280 is not crucial for the action of endogenous factors that affect the stabilization or degradation of *HLA-G* mRNA since the activity of luciferase does not change significantly upon deletion of the corresponding region in 1Fter-4R constructions. Indeed, recent *in silico* searches for candidate microRNAs that could bind the *HLA-G* 3’UTR [43] have shown that the most relevant microRNAs modulating HLA-G expression do not target this region, whereas many potential target sites occur in the segment upstream of the +3165 position. Moreover, considering the seven *HLA-G* 3’UTR haplotypes evaluated here, the deleted sequence in 1Fter-4R constructions exhibits very limited polymorphism because the +3187 G, +3196 G and +3227 A variants are usually restricted to UTR-1, UTR-2 and UTR-18 respectively. Thus, we cannot exclude that these variants have a specific impact that could have been diluted in the global study. Otherwise, although the +3187 A variant (which is shared by all frequent 3’UTR haplotypes except UTR-1) has been previously associated with lower stability of *HLA-G* mRNA, we found that its presence or absence does not significantly impact the luciferase activity when the 1Fter-2R and 1Fter-4R constructions are used, respectively. This result suggests either that its influence requires the whole mRNA (the position +3187 might modify the tertiary structure of the mRNA, exposing the AU-rich motif sites to degradation enzymes) or that putative endogenous factors that degrade mRNA are absent in the cells that we used, whatever their status of HLA-G expression might be. Interestingly, there is a good concordance between data from the present work and data recently obtained *ex vivo* by associating amounts of plasmatic sHLA-G and *HLA-G* 3’UTR polymorphisms from a large cohort of healthy Brazilian and French donors [5]. Indeed, luciferase gene expression was more intensely downregulated by UTRs with the 14 bp insertion allele than by UTRs with the 14 bp deletion allele, a result that corroborates with the observation that individuals presenting the 14 bp Del/Del genotype had significantly higher levels of sHLA-G as compared to individuals with the 14 bp In/In genotype. Among the mechanisms that could explain this observation, the 14-base sequence contains an AUUUG motif that might have an AU-pentamer–like effect on RNA decay [44]. Otherwise, *in silico* analyses have strongly suggested that the 14-base sequence could be a potential target site for microRNAs with the ability to regulate HLA-G expression [43]. Focusing on 3’UTR haplotypes, UTR-1 is classified as high plasma sHLA-G producer and is prone to a lower impact from endogenous factors in the present study. In contrast, UTR-5 and UTR-7 are classified as low producers of plasma sHLA-G and are demonstrated to be predisposed to a higher impact from endogenous factors herein. Likewise, UTR-2, UTR-3, UTR-4 and UTR-6/-18 are classified as intermediate producers of plasma sHLA-G and are prone to medium impact from endogenous factors in the present study. Nonetheless, apparently contradictory results have been previously obtained *ex vivo* [45, 46]: the UTR-5 haplotype was associated with high sHLA-G expression and the UTR-2 haplotype was associated with low sHLA-G expression. The controversy concerns mainly UTR-5 and suggests that polymorphisms of the promoter region associated with this UTR haplotype should be focused [18]. Moreover, the methodology used for ELISA should also be considered, since we used an in-house ELISA instead of a commercial one. It is noteworthy that two recently published median-joining networks of *HLA-G* 3’UTR haplotypes that consider both the frequency and the number of mutations separating each haplotypes [25, 47], have shown that UTR-1 (associated with higher luciferase activity herein), and UTR-5/-7 (associated with lower luciferase activity herein) are the most distant ones.

From a mechanistic point of view, a panel of candidate microRNAs targeting UTR-1, UTR-2, UTR-3, UTR-4, UTR-5, UTR-6/-18 and UTR-7, the most frequent haplotypes has been recently inferred by using three independent algorithms: RNAhybrid, miRanda and PITA [43]. Although we are not aware that the predicted microRNAs actually exist within the cellular environment of each of the cell lines used here, some of these microRNAs could bind to non-polymorphic target sequences. Hence, if these microRNAs were present, they would downregulate the luciferase activity irrespective of the 3’UTR sequence used, whereas other microRNAs would only act depending on the presence of specific variable sites. It has been previously shown that miR-133a regulates HLA-G expression through a non-polymorphic binding site whereas miR-148a, miR-148b, and miR-152 influence HLA-G expression depending on the SNP present at position +3142. More recently miR-628-5p and miR-548q have been demonstrated to target the segment encompassing the variable sites +3003 and +3010 [24]. Notably, there is no consensus about how +3142 SNP [22, 33] impacts on the binding profile of microRNAs, and we have not found a significant effect of the +3035 C-T polymorphism, which suggest that the influence of a genetic background should be considered in a specific microRNA environment.

Regarding UTR-2 response (intermediate downregulation of the luciferase activity) as compared to the response of UTR-5 and UTR-7 (strong downregulation of the luciferase activity) *in vitro*, the lack of significant changes in luciferase activity following the mutagenesis of UTR-5 and UTR-7 at +3035 suggests that other variable sites could be involved. Likewise, we can hypothesize that each haplotype-specific nucleotide variation could influence the level of responses observed with UTR-3, UTR-4 and UTR-18, which all share the 14 bp In variant with UTR-1. In addition, heterogeneous nuclear ribonucleoprotein R (HNRNPR) was recently demonstrated to bind both *MHC classical class I* and *HLA-G* 3’UTR, and was shown to stabilize these *HLA* mRNAs [48]. Although the binding site of HNRNPR is unknown it is thus possible that 3’UTR polymorphism might affect the enhancer activity, more particularly with UTR-5 and UTR-7.

Otherwise, regarding the whole 3’UTR haplotypes effect on luciferase activity in each cell line separately, there are significant differences except for FON as compared to U251MG cells. Therefore, the intensity of the luciferase activity depends on the cellular microenvironment. Because the HLA-G expression involves both transcriptional and post-transcriptional mechanisms [7], a differential contribution of these regulation mechanisms might occur according to the cell type.

Interestingly, we have to point out some unexpected observations (at least at a first glance) regarding 3’UTR haplotypes and the level of HLA-G expression in each cell line. UTR-7, which is theoretically associated with lower HLA-G expression, exists in the HLA-G+ JEG-3 cell line only. UTR-2, which is theoretically associated with intermediate level of HLA-G expression, also exists in JEG-3, and is present in the homozygous state in FON+ cells. Therefore, the presence of at least one UTR-2 haplotype might be linked with levels of HLA-G expression in both cell lines. Besides, the UTR-2/UTR-2 genotype might contribute to higher HLA-G expression in FON+ than in JEG-3 cells (S1 Fig), together with the lower effect of endogenous factors on the *HLA-G* 3’UTR in FON+ cells as compared to JEG-3 cells (Fig 2B). Another mechanism to consider is the partial methylation of *HLA-G* promoter that we had previously demonstrated in JEG-3, but which was not observed in FON+ cells [36]. However the question is still opened concerning the methylated allele which could be the one with the UTR-7 haplotype. Moreover and surprisingly, UTR1, which is theoretically associated with higher HLA-G expression, exists in the HLA-G- cell line (HLA-G mRNA relative to JEG-3: ~1.10^−4^) exclusively, whilst UTR-2 and UTR-4, which are theoretically associated with intermediate HLA-G expression, occur in M8 and U251MG, respectively. We have previously showed that the M8 *HLA-G* promoter is strongly methylated [36] and that demethylation treatment upregulates *HLA-G* transcription in M8 [16, 36] and U251MG cells [37, 49]. This result first suggests that the *HLA-G* gene is functional in these cell lines especially because U251MG-treated cells may produce HLA-G protein [37, 49]. Second, in the present work we have tested the impact of *HLA-G* 3’UTR polymorphism irrespective of modifications in chromatin, and we have focused on post-transcriptional mechanisms only. Given the lowest responses of 3’UTR in M8 cells (Fig 5A), mechanisms acting at the transcriptional level might predominate in these cells. On the other hand, endogenous factors acting on transfected 3’UTR are mobilized in both HLA-G + cells JEG-3 and FON, and in HLA-G- U251MG cells, suggesting that endogenous expression of HLA-G has little or no influence on luciferase reporter assays. Moreover, this result indicates that steady-state levels of HLA-G transcripts in the HLA-G+ cells we used result from a transcriptional activity that counterbalances the repressive action of post-transcriptional mechanisms. As a first step to examine these processes we are currently investigating the pattern of microRNAs in each cell line used in the present study.

In conclusion, we had previously reported that *HLA-G* 3’UTR haplotypes might have a predictive value regarding the genetic predisposition to express different levels of sHLA-G. In agreement, several associations between specific *HLA-G* 3’UTR haplotypes and pathological situations where HLA-G expression is relevant have been identified. For instance, according to our results, UTR-5 and UTR-7 frequencies are higher in recurrent pregnancy loss [50], whereas UTR-1 has been associated with reduced risk of recurrent pregnancy loss [51]. Besides, 3’UTR variants have been identified as potential prognostic biomarkers to determine susceptibility to infections [52, 53],the success of transplantation [54], and the outcome of cancer [55] and autoimmune diseases [56]. Even if we cannot neglect the influence of the *HLA-G* 5’URR haplotypes that are in linkage disequilibrium with 3’UTR [18], here we demonstrated that 3’UTR variants are directly involved in the differential response to cellular endogenous factors and are therefore potential target sites to consider for the design of novel therapeutic approaches.

## Supporting Information

S1 Fig Comparison of steady-state levels of HLA-G mRNAs in JEG-3, M8, U251MG and FON+ cells. One representative Real time RT-PCR analysis performed in triplicate targeting all the HLA-G mRNAs. Results are compared to levels of HLA-G mRNAs in JEG-3 which were assigned a value of 1. The results agree with previously published data.
(TIF)

S1 Table Normalized Luciferase expressions obtained with each HLA-G 3’UTR constructions transfected into HLA-G+ and HLA-G- cell lines. Mean expression, Standard Deviation (SD), and n = sample sizes, are indicated.
(DOCX)
