# Supplementary figures and images for: Haplotypes of the HLA-G 3’ Untranslated Region Respond to Endogenous Factors of HLA-G+ and HLA-G- Cell Lines Differentially
Source: PLoS One. 2017 Jan 3;12(1):e0169032. doi: 10.1371/journal.pone.0169032 (PMC5207740; doi:10.1371/journal.pone.0169032)

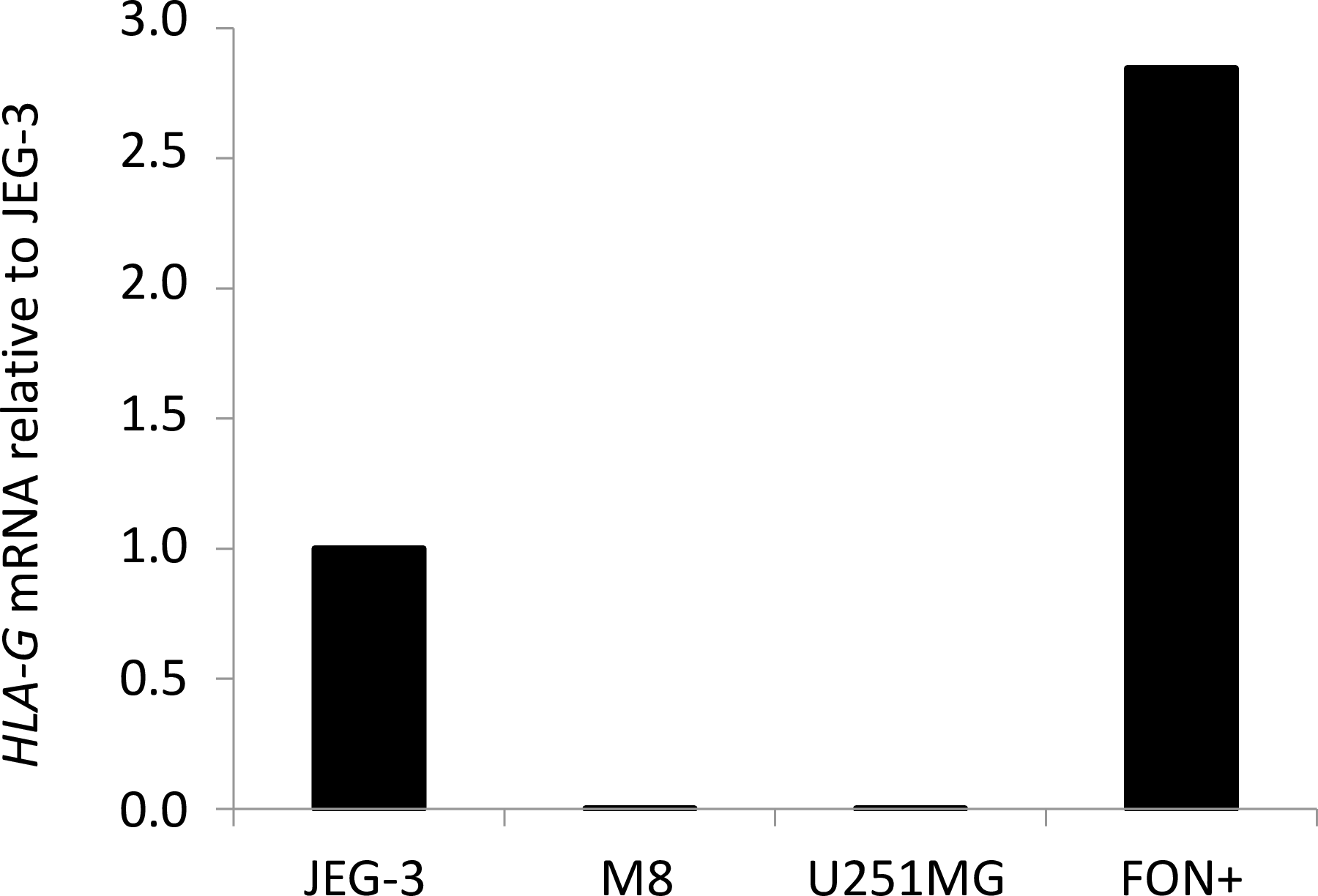

Supplement: S1 Fig — One representative Real time RT-PCR analysis performed in triplicate targeting all the HLA-G mRNAs. Results are compared to levels of HLA-G mRNAs in JEG-3 which were assigned a value of 1. The results agree with previously published data. (TIF) [file pone.0169032.s001.tif]
