# Supplementary material for: Haplotypes of the HLA-G 3’ Untranslated Region Respond to Endogenous Factors of HLA-G+ and HLA-G- Cell Lines Differentially
Source: PLoS One. 2017 Jan 3;12(1):e0169032. doi: 10.1371/journal.pone.0169032 (PMC5207740; doi:10.1371/journal.pone.0169032)
Supplement: S1 Table — Mean expression, Standard Deviation (SD), and n = sample sizes, are indicated. (DOCX) [file pone.0169032.s002.docx]

**Supplemental table 1**  : Normalized Luciferase expressions obtained with each HLA-G 3’UTR constructions transfected into HLA-G+ and HLA-G- cell lines.

|  | HLA-G+ | | | | | HLA-G- | | | | |  |
| --- | --- | --- | --- | --- | --- | --- | --- | --- | --- | --- | --- |
| Cell Lines | JEG-3 | | FON+ | | Both | M8 | | U251MG | | Both | All |
| Constructions | 1Fter2R | 1Fter4R | 1Fter2R | 1Fter4R | Both | 1Fter2R | 1Fter4R | 1Fter2R | 1Fter4R | Both | Both |
| UTR-1 | 0.1750 (0.0363) *n* = 6 | 0.1348 (0.0403) *n* = 6 | 0.4801 (0.0729) *n* = 8 | 0.5212 (0.1868) *n* = 8 | 0.3525 (0.2043) *n* = 28 | 0.3728 (0.2845) *n* = 6 | 0.4493 (0.1575) *n* = 8 | 0.1893 (0.0049) *n* = 6 | 0.1969 (0.0150) *n* = 6 | 0.3134 (0.1921) *n* = 26 | 0.3337 (0.1976) *n* = 54 |
| UTR-2 | 0.0833 (0.0135) *n* = 6 | 0.0818 (0.0278) *n* = 6 | 0.3073 (0.1410) *n* = 6 | 0.2143 (0.0589) *n* = 8 | 0.1750 (0.1181) *n* = 26 | 0.3392 (0.2525) *n* = 8 | 0.3625 (0.1237) *n* = 8 | 0.1728 (0.0394) *n* = 6 | 0.1679 (0.0303) *n* = 6 | 0.2735 (0.1712) *n* = 28 | 0.2260 (0.1549) *n* = 54 |
| UTR-3 | 0.1580 (0.0274) *n* = 6 | 0.1449 (0.0708) *n* = 6 | 0.2408 (0.1018) *n* = 6 | 0.1348 (0.0897) *n* = 8 | 0.1670 (0.0852) *n* = 26 | 0.4806 (0.1848) *n* = 8 | 0.2854 (0.0753) *n* = 4 | 0.2633 (0.0643) *n* = 6 | 0.2312 (0.0424) *n* = 6 | 0.3314 (0.1561) *n* = 24 | 0.2459 (0.1484) *n* = 50 |
| UTR-4 | 0.0813 (0.0089) *n* = 6 | 0.1341 (0.0608) *n* = 6 | 0.0991 (0.0242) *n* = 8 | 0.3662 (0.0565) *n* = 8 | 0.1791 (0.1285) *n* = 28 | 0.5299 (0.3186) *n* = 8 | 0.4363 (0.1290) *n* = 6 | 0.1804 (0.0389) *n* = 5 | 0.2338 (0.1074) *n* = 6 | 0.3665 (0.2397) *n* = 25 | 0.2675 (0.2098) *n* = 53 |
| UTR-5 | 0.0508 (0.0162) *n* = 6 | 0.0706 (0.0206) *n* = 6 | 0.0692 (0.0235) *n* = 8 | 0.1053 (0.0686) *n* = 8 | 0.0758 (0.0436) *n* = 28 | 0.1660 (0.0813) *n* = 8 | 0.3263 (0.1111) *n* = 6 | 0.0602 (0.0262) *n* = 6 | 0.1669 (0.0392) *n* = 6 | 0.1788 (0.1160) *n* = 26 | 0.1254 (0.1000) *n* = 54 |
| UTR-18 | 0.0874 (0.0135) *n* = 6 | 0.0919 (0.0264) *n* = 6 | 0.2666 (0.1245) *n* = 8 | 0.2221 (0.1853) *n* = 8 | 0.1780 (0.1395) *n* = 28 | 0.2347 (0.0501) *n* = 8 | 0.4055 (0.3190) *n* = 8 | 0.3068 (0.0649) *n* = 6 | 0.2135 (0.0282) *n* = 6 | 0.2944 (0.1848) *n* = 28 | 0.2362 (0.1725) *n* = 56 |
| UTR-7 | 0.0493 (0.0215) *n* = 6 | 0.0544 (0.0086) *n* = 6 | 0.0549 (0.0271) *n* = 8 | 0.0697 (0.0370) *n* = 8 | 0.0578 (0.0266) *n* = 28 | 0.3519 (0.1428) *n* = 8 | 0.4335 (0.2287) *n* = 8 | 0.1283 (0.0136) *n* = 6 | 0.1295 (0.0421) *n* = 6 | 0.2796 (0.1946) *n* = 28 | 0.1687 (0.1774) *n* = 56 |

Mean expression, Standard Deviation (SD), and n=sample sizes, are indicated.
